# Supplementary material for: HIVprotI: an integrated web based platform for prediction and design of HIV proteins inhibitors
Source: J Cheminform. 2018 Mar 9;10:12. doi: 10.1186/s13321-018-0266-y (PMC5845081; doi:10.1186/s13321-018-0266-y)
Supplement: Supplementary file 2 — Additional file 2. Source code of HIVProtI web server. [file 13321_2018_266_MOESM2_ESM.zip › HIVprotI_Source-code/hivprotI/weka-3-6-6/doc/weka/classifiers/RandomizableSingleClassifierEnhancer.html]

RandomizableSingleClassifierEnhancer


---


|  |  |  |
| --- | --- | --- |
| **Overview** \| **Package** \| **Class** \| **Tree** \| **Deprecated** \| **Index** \| **Help** \| **Weka's home** | |  |
| **PREV CLASS**   **NEXT CLASS** | **FRAMES**    **NO FRAMES**     **All Classes** |  |
| SUMMARY: NESTED \| FIELD \| CONSTR \| METHOD | DETAIL: FIELD \| CONSTR \| METHOD |  |


---


## weka.classifiers Class RandomizableSingleClassifierEnhancer

```
java.lang.Object
  weka.classifiers.Classifier
      weka.classifiers.SingleClassifierEnhancer
          weka.classifiers.RandomizableSingleClassifierEnhancer
```

**All Implemented Interfaces:**: java.io.Serializable, java.lang.Cloneable, CapabilitiesHandler, OptionHandler, Randomizable, RevisionHandler

**Direct Known Subclasses:**: ClassBalancedND, CostSensitiveClassifier, CVParameterSelection, Dagging, DataNearBalancedND, GridSearch, MetaCost, MultiClassClassifier, ND, RacedIncrementalLogitBoost, ThresholdSelector

---

``` public abstract class RandomizableSingleClassifierEnhancer extends SingleClassifierEnhancer implements Randomizable ```

Abstract utility class for handling settings common to randomizable
meta classifiers that build an ensemble from a single base learner.

**Version:**
:   $Revision: 1.4 $

**Author:**
:   Eibe Frank (eibe@cs.waikato.ac.nz)

**See Also:**: Serialized Form

---

**Constructor Summary**

`RandomizableSingleClassifierEnhancer()`


| **Method Summary** | |
| --- | --- |
| `java.lang.String[]` | `getOptions()`             Gets the current settings of the classifier. |
| `int` | `getSeed()`             Gets the seed for the random number generations |
| `java.util.Enumeration` | `listOptions()`             Returns an enumeration describing the available options. |
| `java.lang.String` | `seedTipText()`             Returns the tip text for this property |
| `void` | `setOptions(java.lang.String[] options)`             Parses a given list of options. |
| `void` | `setSeed(int seed)`             Set the seed for random number generation. |

**Methods inherited from class weka.classifiers.SingleClassifierEnhancer**

`classifierTipText, getCapabilities, getClassifier, setClassifier`

**Methods inherited from class weka.classifiers.Classifier**

`buildClassifier, classifyInstance, debugTipText, distributionForInstance, forName, getDebug, getRevision, makeCopies, makeCopy, setDebug`

**Methods inherited from class java.lang.Object**

`equals, getClass, hashCode, notify, notifyAll, toString, wait, wait, wait`

**Constructor Detail**

### RandomizableSingleClassifierEnhancer

```
public RandomizableSingleClassifierEnhancer()
```


**Method Detail**

### listOptions

```
public java.util.Enumeration listOptions()
```

:   Returns an enumeration describing the available options.

    :   **Specified by:**: `listOptions` in interface `OptionHandler` **Overrides:**: `listOptions` in class `SingleClassifierEnhancer`
    :   **Returns:**: an enumeration of all the available options.

---


### setOptions

```
public void setOptions(java.lang.String[] options)
                throws java.lang.Exception
```

:   Parses a given list of options. Valid options are:

    -W classname   
    Specify the full class name of the base learner.

    -I num   
    Set the number of iterations (default 10).

    -S num   
    Set the random number seed (default 1).

    Options after -- are passed to the designated classifier.

    :   **Specified by:**: `setOptions` in interface `OptionHandler` **Overrides:**: `setOptions` in class `SingleClassifierEnhancer`
    :   **Parameters:**: `options` - the list of options as an array of strings **Throws:**: `java.lang.Exception` - if an option is not supported

---


### getOptions

```
public java.lang.String[] getOptions()
```

:   Gets the current settings of the classifier.

    :   **Specified by:**: `getOptions` in interface `OptionHandler` **Overrides:**: `getOptions` in class `SingleClassifierEnhancer`
    :   **Returns:**: an array of strings suitable for passing to setOptions

---


### seedTipText

```
public java.lang.String seedTipText()
```

:   Returns the tip text for this property

    :   **Returns:**: tip text for this property suitable for displaying in the explorer/experimenter gui

---


### setSeed

```
public void setSeed(int seed)
```

:   Set the seed for random number generation.

    :   **Specified by:**: `setSeed` in interface `Randomizable`
    :   **Parameters:**: `seed` - the seed

---


### getSeed

```
public int getSeed()
```

:   Gets the seed for the random number generations

    :   **Specified by:**: `getSeed` in interface `Randomizable`
    :   **Returns:**: the seed for the random number generation


---


|  |  |  |
| --- | --- | --- |
| **Overview** \| **Package** \| **Class** \| **Tree** \| **Deprecated** \| **Index** \| **Help** \| **Weka's home** | |  |
| **PREV CLASS**   **NEXT CLASS** | **FRAMES**    **NO FRAMES**     **All Classes** |  |
| SUMMARY: NESTED \| FIELD \| CONSTR \| METHOD | DETAIL: FIELD \| CONSTR \| METHOD |  |


---
